# Plastid phylogenomics and morphological character evolution of Chloridoideae (Poaceae)

**DOI:** 10.3389/fpls.2022.1002724

**Published:** 2022-11-02

**Authors:** Rong Wang, Xue-Jie Zhang, Xiu-Xiu Guo, Yan Xing, Xiao-Jian Qu, Shou-Jin Fan

**Affiliations:** ^1^ Shandong Provincial Key Laboratory of Plant Stress Research, College of Life Sciences, Shandong Normal University, Jinan, China; ^2^ Innovative Institute of Chinese Medicine and Pharmacy, Shandong University of Traditional Chinese Medicine, Jinan, China

**Keywords:** Chloridoideae, plastome feature, plastid phylogenomics, morphological character evolution, divergence time estimations

## Abstract

Chloridoideae is one of the largest subfamilies of Poaceae, containing many species of great economic and ecological value; however, phylogenetic relationships among the subtribes and genera of Cynodonteae are controversial. In the present study, we combined 111 plastomes representing all five tribes, including 25 newly sequenced plastomes that are mostly from Cynodonteae. Phylogenetic analyses supported the five monophyletic tribes of Chloridoideae, including Centropodieae, Triraphideae, Eragrostideae, Zoysieae and Cynodonteae. Simultaneously, nine monophyletic lineages were revealed in Cynodonteae: supersubtribe Boutelouodinae, subtribes Tripogoninae, Aeluropodinae, Eleusininae, Dactylocteniinae, supersubtribe Gouiniodinae, *Cleistogenes* and *Orinus*, and subtribe Triodiinae. Within the tribe of Cynodonteae, the basal lineage is supersubtribe Boutelouodinae and Tripogoninae is sister to the remaining lineages. The clade formed of Aeluropodinae and Eleusininae is sister to the clade composed of Dactylocteniinae, supersubtribe Gouiniodinae, *Cleistogenes* and *Orinus*, and subtribe Triodiinae. The clade comprising Dactylocteniinae and supersubtribe Gouiniodinae is sister to the clade comprising *Cleistogenes*, *Orinus*, and Triodiinae. *Acrachne* is a genus within Eleusininae but not within Dactylocteniinae. Molecular evidence determined that *Diplachne* is not clustered with *Leptochloa*, which indicated that *Diplachne* should not be combined into *Leptochloa*. *Cleistogenes* is sister to a clade composed of *Orinus* and *Triodia*, whereas the recently proposed subtribe Orininae was not supported. Cynodonteae was estimated to have experienced rapid divergence within a short period, which could be a major obstacle in resolving its phylogenetic relationships. Ancestral state reconstructions of morphological characters showed that the most recent common ancestor (MRCA) of Chloridoideae has a panicle, multiple florets in each spikelet, the peaked type of stomatal subsidiary cells, and a saddle-shaped phytoliths, while the ancestral morphological characters of Cynodonteae are the panicle, peaked type of stomatal subsidiary cells, sharp-cap cell typed and equal-base-cell microhair, and square-shaped phytoliths. Overall, plastome phylogenomics provides new insights into the phylogenetic relationships and morphological character evolution of Chloridoideae.

## Introduction

Chloridoideae (Poaceae, Poales) was established by Beilschmied (1833). This subfamily is comprised of more than 1400 species in approximately 140 genera all around the world, which are mainly distributed in arid tropical and subtropical regions (Watson and Dallwitz, 1992; Clayton et al., 2008). Many species in Chloridoideae have important economic and application value. Some species of this subfamily are important crops, such as *Eragrostis tef* (D’Andrea, 2008; Zhu, 2018; Gelaw and Qureshi, 2020) and *Eleusine coracana* (Chandrashekar, 2010; Devi et al., 2014; Chandra et al., 2016). There are also some common landscaping plants, such as bermuda grass (*Cynodon dactylon*) (Taliaferro, 1995) and Janpanese lawn grass (*Zoysia japonica*) (Croce et al., 2001). In Chloridoideae, the majority of species use the C_4_ photosythetic pathway, so this family is an important group for studying the evolutionary transition from C_3_ to C_4_ photosynthesis in grasses (GPWG II, 2012). Microhairs have been observed in many subfamilies of Poaceae as a micromorphological characteristic of the leaf epidermis of grasses, but only function as salt glands in Chloridoideae (Amarasinghe and Watson, 1989; Ramadan, 2001; Chen et al., 2003; Kobayashi et al., 2007). It is suggested that salt glands play important roles in secretion (Marcum et al., 1998). Chloridoideae is useful for studying tolerance to different kinds of abiotic stresses in grasses (Marcum, 1999; Subudhi and Baisakh, 2011; Huang et al., 2017; Wang et al., 2020).

Chloridoideae shows great morphological diversity, especially in inflorescence, spikelet and micromorphology (Liu et al., 2010; Pilatti et al., 2018; Pilatti et al., 2019). These morphological characters are also important taxonomic features to classify Chloridoideae taxa (Clayton, 1982). An inflorescence consists of a group of flowers or clusters of flowers arranged on a stem. Inflorescence of Chloridoideae ranged from loose or dense panicles with a large number of spikelets to simple, single-spikelet inflorescences (Pilatti et al., 2018). It is difficult to analyze the inflorescence evolution pattern because of the complexity (Doust and Kellogg, 2002). Spikelets are novel and developmentally integrated structure in Poaceae (Wang et al., 2022). They are highly specialized structure and are basic units of grass inflorescences (Wang et al., 2022). In Chloridoideae, spikelets may be single- or many-flowered and they may be composed of unisexual, bisexual or both types of flowers (Watson and Dallwitz, 1992; Kinney et al., 2008). In addition to macromorphological characters, micromorphological characters are also very important in Chloridoideae classification. It is reported that patterns of the lemma micromorphology are a useful tool in taxonomy of the Middle Asian *Eragrostis* species (Poaceae) (Wróbel et al., 2017). Grasses have a unique stomatal structure with elongated dumbbell-shaped guard cells and two subsidiary cells (Stebbins and Shah, 1960). Microhairs are bicellular strctures in most taxa of Poaceae (Marcum et al., 1998; Marcum, 1999). A bicellular microhair is composed of a basal cell and a cap cell (Oross and Thomson, 1982b; Barhoumi et al., 2008). Morphology of stomata and microhairs may play important roles in stress tolerance in Poaceae. Silica entered into plants through roots and deposited as inclusions within the cells, they are usually termed as phytoliths or silica bodies. Phytoliths have proved to be a potential tool in palaeoecological studies and Chloridoideae taxa identification (Jattisha and Sabu, 2012). The study of these morphological characters will eventually contribute to functional study.

Molecular phylogenetic studies showed that Chloridoideae is a monophyletic group within the PACMAD clade and is sister to the subfamily Danthonioideae (GPWG II, 2012; Soreng et al., 2015; Soreng et al., 2017; Saarela et al., 2018). It is difficult to elucidate phylogenetic relationships within Chloridoideae. In the most recent classification study of Chloridoideae, Chlorodoideae was classified into five tribes, including Centropodieae, Triraphideae, Eragrostideae, Zoysieae, and Cynodonteae (Peterson et al., 2011; Stull et al., 2015; Soreng et al., 2017). Centropodieae was newly established based on the nature of monophyly and its photosynthetic mode (Peterson et al., 2011). The phylogenetic relationships among the five tribes have been confirmed in phylogenetic studies (Peterson et al., 2011; Peterson et al., 2012; Duvall et al., 2016; Zhang et al., 2016; Peterson et al., 2017). Centropodieae and Triraphideae are successively diverged groups of Chloridoideae. Zoysieae and Cynodonteae are sister groups, and Eragrostideae is the sister clade to the clade composed of Zoysieae and Cynodonteae.

Cynodonteae is recognized as a derived and species-rich group of Chloridoideae. Peterson et al. (2010) recognized 13 monophyletic subtribes of Cynodonteae in a phylogenetic tree based on plastid and ITS sequences, including Aeluropodinae, Triodiinae, Orcuttiinae, Tridentinae, Eleusininae, Tripogoninae, Pappophorinae, Traginae, Hilariinae, Monanthochloinae, Boutelouinae, Scleropogoninae and Muhlenbergiinae. Soreng et al. (2015) established six subtribes (Gouiniinae, Cteniinae, Trichoneurinae, Perotidinae, Farragininae, and Gymnopogon) and discarded Tridentinae when compared with the classification of Peterson et al. (2010). In the most recent classification study, Soreng et al. (2017) recognized four new subtribes (Dactylocteniinae, Orininae, Hubbardochloinae, and Zaqiqahinae) and established two supersubtribes (supersubtribe Bouteloudinae and supersubtribe Gouiniodinae). Supersubtribe Bouteloudinae includes Boutelouinae, Hilariinae, Monanthochloinae, Muhlenbergiinae, Scleropogoninae and Traginae. Supersubtribe Gouiniodinae includes Cteniinae, Farragininae, Gouiniinae, Hubbardochloinae, Perotidinae, Trichoneurinae and Zaqiqahinae. Phylogenetic relationships among subtribes of Cynodonteae are not well resolved, and contain weakly supported and conflicting relationships (Peterson et al., 2010). The topology of intersubtribe phylogenetic trees based on ITS, plastid and combined sequences are similar, but there are still some incongruences, such as between Aeluropodininae and Eleusininae (Soreng et al., 2015; Soreng et al., 2017).

Chloroplasts are important organelles in plant cells (Leister, 2003; Lancien et al., 2006). The plastomes of angiosperms usually have a typical quadripartite structure with two inverted repeat (IR) regions separated by a large single-copy (LSC) region and a small single-copy (SSC) region (Jansen and Ruhlman, 2012). (Hubbard, 1936; Stull et al., 2015; Attigala et al., 2016; Uribe-Convers et al., 2017). With the rapid development of next-generation sequencing technology, plastomes have been increasingly adopted in grass phylogenetic studies (Liu et al., 2020a; Orton et al., 2021). For Chloridoideae, Duvall et al. (2016) applied plastome data to resolve the phylogenetic relationships of tribes and genera. The plastomes of Eragrostideae were compared and used to study intergeneric phylogenetic relationships (Somaratne et al., 2019; Teshome et al., 2020; Liu et al., 2021a). Wang et al. (2021) provided new insights into the inter- and intrageneric phylogenetic relationships of *Cleistogenes* based on plastome phylogenomics. In addition, molecular dating analyses based on plastome phylogeny revealed the dispersal path of tetraploid and hexaploid lineages of *Spartina* (Rousseau-Gueutin et al., 2015).

In this study, we newly sequenced 25 Chloridoideae plastomes. The purpose of this study was to 1) explore the phylogenetic relationships among tribes of Chloridoideae, especially among the subtribes and genera of Cynodonteae; 2) discuss the causes of the complex intersubtribe relationships of Cynodonteae; and 3) reconstruct the ancestral morphological character of Chloridoideae and Cynodonteae.

## Materials and methods

### Taxon sampling, DNA extraction, and sequencing

Taxon sampling was guided by the recent classification of Poaceae (Soreng et al., 2015; Soreng et al., 2017). A total of 111 plastomes representing 111 taxa (including three outgroups) were used as plant materials in the present study. These taxa belong to five tribes of Chloridoideae. Among them, 25 plastomes were sequenced in the present study, 15 plastomes were sequenced in Wang et al. (2021), a plastome (*Harpachne harpachnoides*) in Liu et al. (2021a), a plastome (*Eleusine coracana*) in Liu et al. (2021b) and 69 plastomes were downloaded from NCBI (**Table S1**). Voucher information of 25 newly sequenced Chloridoideae taxa is shown in **Table 1**. Total DNA was extracted from dried leaves that were collected in the field. Voucher specimens and silica-dried leaves were stored at the College of Life Sciences, Shandong Normal University (SDNU), Ji’nan, China. Total genomic DNA was extracted using a modified CTAB method (Doyle and Doyle, 1987). DNA quality and concentrations were examined by gel electrophoresis and a NanoDrop 2000 spectrophotometer (Thermo Scientific, Wilmington, USA) to examine the quality and integrity of DNA. Final DNA concentrations of samples over 30 ng/µL could be used for sequencing. Total genomic DNA was used to construct sequence libraries following the manufacturer’s protocol. Paired-end (PE) sequencing libraries were sequenced using the Illumina NovaSeq platform at Novogene (Beijing, China).

**Table 1:** Voucher information of 25 newly sequenced Chloridoideae taxa.

Taxa	Locality	Collection number	Latitude	Longitude	Genebank accession number
*Acrachne racemosa *	Hainan, China	HN01	19°15’N	109°0’E	OM307668
*Bouteloua dactyloides*	Beijing, China	006	40°7’N	116°39’E	OM307669
*Crypsis aculeata*	Shandong, China	9408-008	37°20’N	118°27’E	OM307670
*Cynodon radiatus*	Hainan, China	HN02	19°57’N	110°19’E	OM307671
*Desmostachya bipinnata*	Hainan, China	HN03	19°7’N	108°39’E	OM307672
*Dinebra retroflexa*	Shandong, China	46	36°7’N	120°25’E	OM307673
*Diplachne fusca*	Shandong, China	2242	37°24’N	120°46’E	OM307674
*Enneapogon desvauxii*	Inner mogolia, China	046	40°51’N	111°35’E	OM307675
*Enteropogon dolichostachyus*	Yunnan, China	004	24°48’N	100°33’E	OM307676
*Leptochloa chinensis*	Guangdong, China	123	22°42’N	111°57’E	OM307677
*Leptochloa panicea*	Shandong, China	061018	36°39’N	117°2’E	OM307678
*Lepturus repens*	Hainan, China	HN04	19°7’N	108°39’E	OM307679
*Microchloa indica*	Yunnan, China	195	25°56’N	100°24’’E	OM307680
*Muhlenbergia huegelii*	Hebei, China	HB06	40°31’N	117°30’E	OM307681
*Muhlenbergia japonica*	Shandong, China	006	36°21’N	118°2’’E	OM307682
*Muhlenbergia ramosa*	Shandong, China	196	35°59’N	118°33’’E	OM307683
*Orinus thoroldii*	Tibet, China	XZ001	29°20’N	88°58’E	OM307684
*Perotis hordeiformis*	Hainan, China	HN05	18°25’N	109°51’E	OM307685
*Perotis indica*	Hainan, China	HN06	19°8’N	108°41’E	OM307686
*Perotis rara*	Hainan, China	HN07	19°25’N	108°50’E	OM307687
*Sporobolus diander*	Guangdong, China	06	21°10’N	110°19’	OM307688
*Sporobolus fertilis*	Yunnan, China	17	24°40’N	102°19’	OM307689
*Sporobolus virginicus*	Guangdong, China	18	21°7’N	110°18’	OM307690
*Tragus berteronianus*	Liaoning, China	218	41°29’N	120°25’E	OM307691
*Tragus mongolorum*	Shandong, China	067	36°40’N	117°2’E	OM307692

### Plastome assembly and annotation

Plastomes were assembled using GetOrganelle v1.7.4.1 (Jin et al., 2020) with SPAdes v3.12.0 as the internal assemble (Bankevich et al., 2012). The k-mers were 61, 81, 101 and 121. All paired reads were mapped to assembled plastomes with Bowtie v2.3.2 with the local-sensitive option (-D 15 -R 2 -N 0 -L 20 -i S,1,0.75) to validate the plastomes assembly. PGA (Plastid Genome Annotator) was used for plastome annotation (Qu et al., 2019). During plastome annotation, the plastome of *Amborella trichopoda* was used as a reference. Manual corrections were conducted after annotation using Geneious v9.1.4 (Kearse et al., 2012). It showed that there are some errors exist in the annotation of the plastome available in a public database (Amiryousefi et al., 2018; Abdullah et al., 2021). For a good comparasion, plastome sequences downloaded from the NCBI database should be reannotated using an approach similar to plastomes sequenced in the present study.

### Phylogenetic analysis

Phylogenetic trees were constructed based on three datasets (protein-coding genes, noncoding regions and complete plastomes). Protein-coding genes and noncoding regions were extracted separately using a Perl script. MAFFT v7.313 was applied in sequence alignment (Katoh and Standley, 2013). The maximum likelihood (ML) tree was constructed using RAxML v8.2.10 (Alexandros, 2014) with 1,000 bootstrap replicates and the GTRGAMMA model. The Bayesian inference (BI) tree was constructed using MrBayes v3.2.6 with Markov chain Monte Carlo (MCMC) run for 1,000,000 steps with a random starting tree, and one tree was sampled every 1,000 steps. The first 25% steps were discarded as burn-in. Species tree analysis was performed with ML in RAxML and the multispecies coalescent summary method in ASTRAL v.5.6.3 (Mirarab et al., 2014). The branch was described as strong support when BS≥80 or PP≥0.95; moderate support when BS≥60 or 0.95≥PP≥0.85; and weak support otherwise.

### Divergence time estimations

Divergence time was estimated for each internal node of the phylogenetic tree. A relaxed clock method and penalized likelihood were involved in dating analyses using treePL (Sanderson, 2002; Smith and O’Meara, 2012). A smoothing parameter of 100 was determined using the cross-validation option, and priming was used to determine the best optimization scores. A total of 1,000 ML bootstrap trees with branch lengths were generated using RAxML (Alexandros, 2014). The maximum age of Centropodieae and the core Chloridoideae crown node was assigned as 43 million years ago (Ma) (Rousseau-Gueutin et al., 2015). The minimum age of Centropodieae and the core Chloridoideae crown node was assigned as 32 Ma (Liu et al., 2011). The minimum age of the Zoysieae and Eragrostideae crown nodes was assigned as 25.3 Ma (Liu et al., 2011). The minimum age of the Zoysieae and Cynodonteae crown nodes was assigned as 22.5 Ma (Vicentini et al., 2008). The minimum and maximum ages for the internal nodes were calculated from dating 1,000 bootstrap trees by using treePL and Tree Annotator v1.8.495 (Drummond et al., 2012).

### Ancestral state reconstruction of Chloridoideae

A total of 51 Chloridoideae taxa were included in this analysis (**Table S3**). Inflorescence and spikelets were observed using Olympus SZ51 (Olympus, Japan). The observation of stomatal subsidiary cells, microhairs and phytoliths on the leaf epidermis was performed on a Hitachi TM3030 (Hitachi, Japan). Mesquite v2.75 (Maddison, 2008) was used to infer the ancestral state reconstructions of Chloridoideae. Ancestral state reconstructions were carried out using the maximum likelihood method with an equal-rate model (Mk1, single rate of transition for both forward and backward change). This analysis was based on the topology of the BI tree. *Centropodia glauca* was used as an outgroup. Characters were unordered and equally weighted. Morphological characters and their state were coded as follows: (a) inflorescence type: panicle (1), raceme (2), panicle composed of spikes (3), panicle composed of racemes (4), spike (5); (b) spikelet type: one floret in each spikelet (1), two florets in each spikelet (2), multiple florets in each spikelet (3); (c) stomatal subsidiary cell shape: peaked type (1), low-domed type (2), flat-top type (3); (d) microhair type: enneapogonoid type (1), long-base cell microhair with sharp-cap cell (2), long-base cell microhair with round-cap cell (3), long-base cell microhair with non-constricted base (4), narrow equal-base-cell microhair (5), well-proportioned equal-base-cell microhair with sharp-cap cell (6), short equal-base-cell microhair with round-cap cell (7); (e) phytoliths: square (1), two-lobed with no obvious rod-like structure (2), short dumbbell type with square lobes (3), four-lobes, cross (4), oval (5), saddle (6), short dumbbell type with round lobes (7), long dumbbell type with round lobes (8).

## Results

### Features of Chloridoideae plastomes

Features of 108 Chloridoideae plastomes were shown in **Table S4**. The Chloridoideae plastomes varied in length from 130,773 bp (*Eragrostis tenellula*) to 138,504 bp (*Distichlis spicata*). They had typical circular quadripartite structures, like those of most angiosperms, consisting of a pair of inverted repeat (IR) regions (19,134-21,225 bp) separated by the LSC regions (77,993 bp-83,456 bp) and the SSC regions (12,302 bp-12,762 bp). The whole GC content of these 109 plastomes were very similar (38.1%-38.5%). In most species, a total of 130-132 genes (110-112 unique genes) were annotated, including 84-86 protein-coding genes (76-78 unique protein-coding genes), 38 tRNA genes and eight rRNA genes. There is a loss of *rps15* in *Eragrostis tenellula*. The gene *trnN*-*GUU* has two copies located in IR regions separately in most species, however, there is another copy located in the LSC region in *Distichlis spicata*. Seven protein-coding genes (*rps19*, *rpl2*, *rpl23*, *ycf2*, *ndhB*, *rps7*, *rps15*), eight tRNA genes (*trnH*-*GUG*, *trnI*-*CAU*, *trnL*-*CAA*, *trnV*-*GAC*, *trnI*-*GAU*, *trnA*-*UGC*, *trnR*-*ACG*, *trnN*-*GUU*) and four rRNA genes (*rrn16*, *rrn23*, *rrn4.5*, *rrn5*) were duplicated in most species. Ten genes contain introns, eight of them (*atpF*, *ndhA*, *ndhB*, *petB*, *petD*, *rpl2*, *rpl16*, *rps16*) have one intron, and two of them (*rps12*, *ycf3*) have two introns.

### Phylogenetic analysis

**Figure 1:**
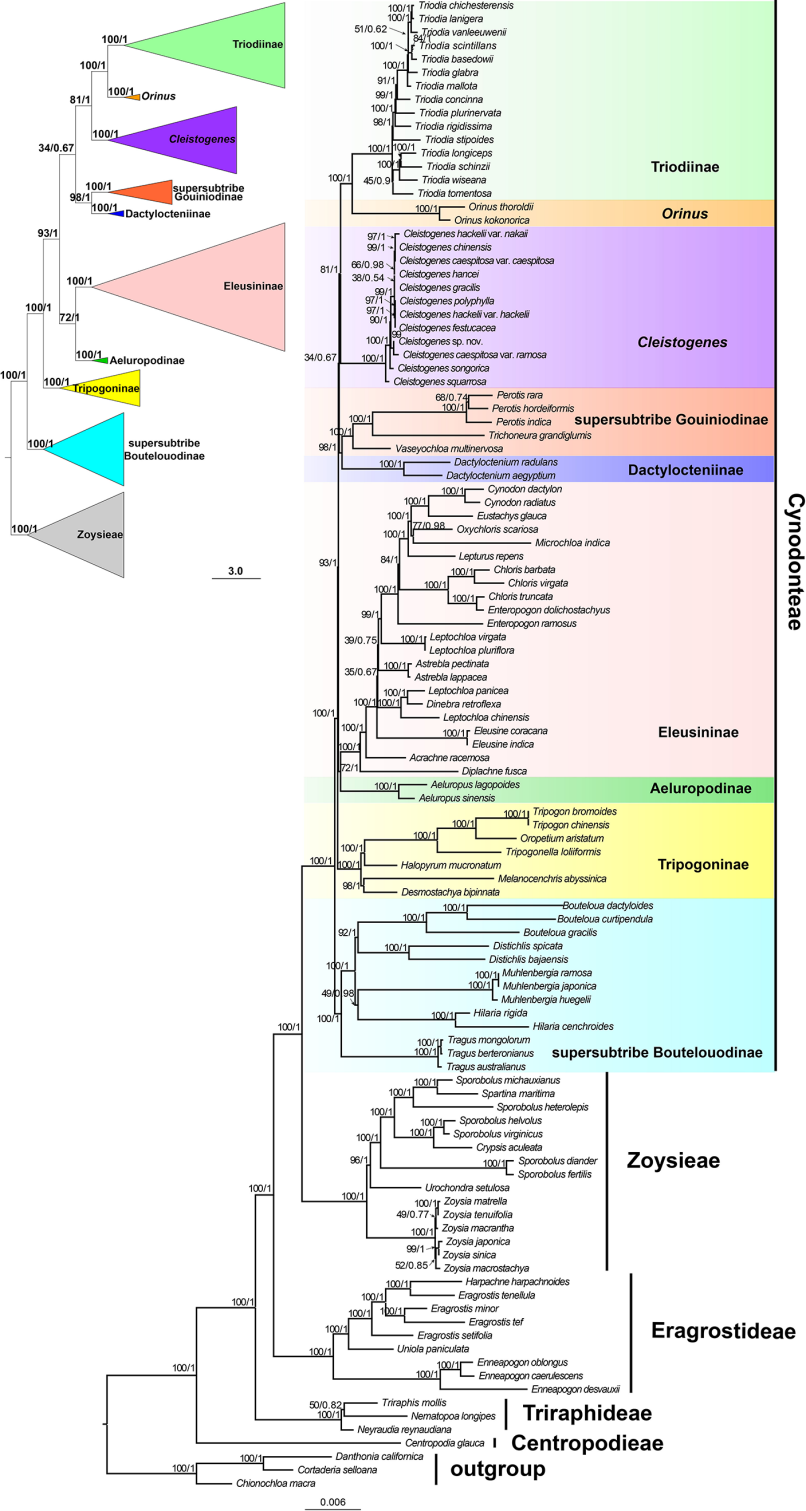
Phylogenetic relationships of Chloridoideae inferred from maximum likelihood (ML) and Bayesian inference (BI) based on 111 complete plastomes (excluding one copy of the inverted repeat (IR)). Support values marked above the branches follow the order bootstrap value (BS)/posterior probability (PP).

**Figure 2:**
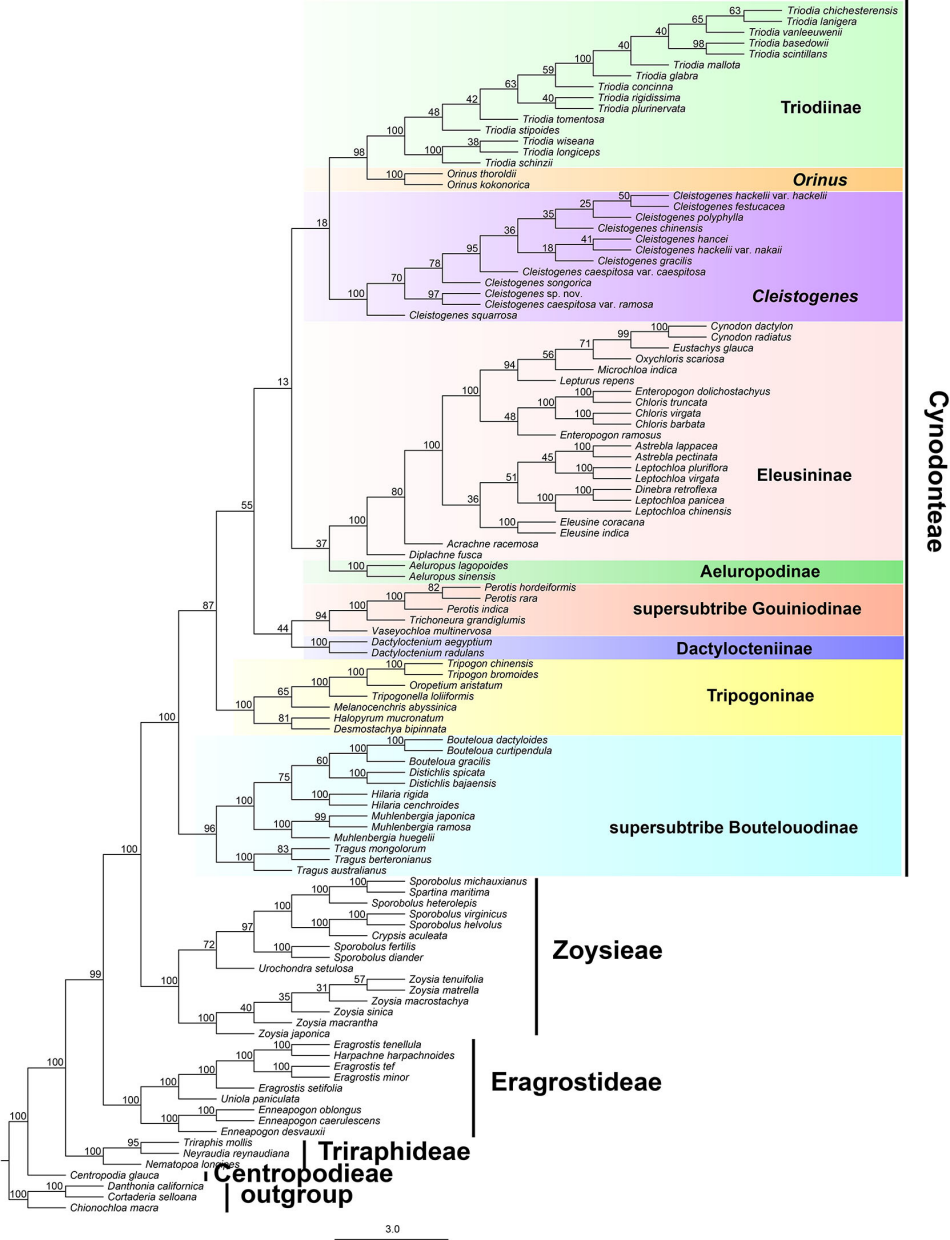
Phylogeny estimated under the multispecies coalescent with ASTRAL from ML gene trees. Values above internodes are bootstrap values (BS).

Maximum likelihood and Bayesian inference phylogenetic analyses using three datasets (protein-coding genes, noncoding regions and complete plastomes) generated identical topologies for Chloridoideae (**Figures 1**, **S1**, **S2**). Five tribes were recovered in the phylogenetic study of Chloridoideae (**Figures 1**, **S1**, **S2**). As shown in **Figure 1**, Cynodonteae and Zoysieae are sister groups with bootstrap (BS) values of 100 and posterior probability (PP) values of 1. The Cynodonteae-Zoysieae clade is sister to Eragrostideae (BS=100, PP=1). The clade composed of Cynodonteae, Zoysieae and Eragrostideae is sister to Triraphideae (BS=100, PP=1). Centropodieae is sister to the clade composed of four other tribes of Chloridoideae (BS=100, PP=1). The monophyly of Cynodonteae is strongly supported (BS=100, PP=1), and a total of nine major lineages are identified within the tribe Cynodonteae (**Figure 1**). For Cynodonteae, the first diverged lineage is supersubtribe Boutelouodinae (BS=100, PP=1), which includes *Tragus*, *Hilaria*, *Muhlenbergia*, *Distichlis* and *Bouteloua*. Tripogoninae is the second-diverged lineage (BS=100, PP=1), which includes *Desmostachya*, *Melanocenchris*, *Halopyrum*, *Tripogonella, Oropetium* and *Tripogon*. The third diverged lineage is Eleusininae and Aeluropodinae (BS=100, PP=1). In Eleusininae, *Diplachne fusca* and *Acrachne racemosa* are the successive early diverging groups. *Leptochloa* is not a monophyletic group. The fourth diverged lineage is the clade composed of Dactylocteniinae and the supersubtribe Gouiniodinae (BS=98, PP=1). The fifth diverged lineage is the clade comprising *Cleistogenes*, *Orinus*, and *Triodia* (BS=81, PP=1). In this clade, *Cleistogenes* is sister to the clade composed of *Orinus* and *Triodia*. The species tree has a similar topology to the ML and BI tree except for the phylogenetic positions of the clade comprising supersubtribe Gouiniodinae and Dactylocteniinae and the clade composed of Eleusininae and Aeluropodinae; however, these two clades have not been resolved (**Figure 2**).

### Divergence time estimations

**Figure 3:**
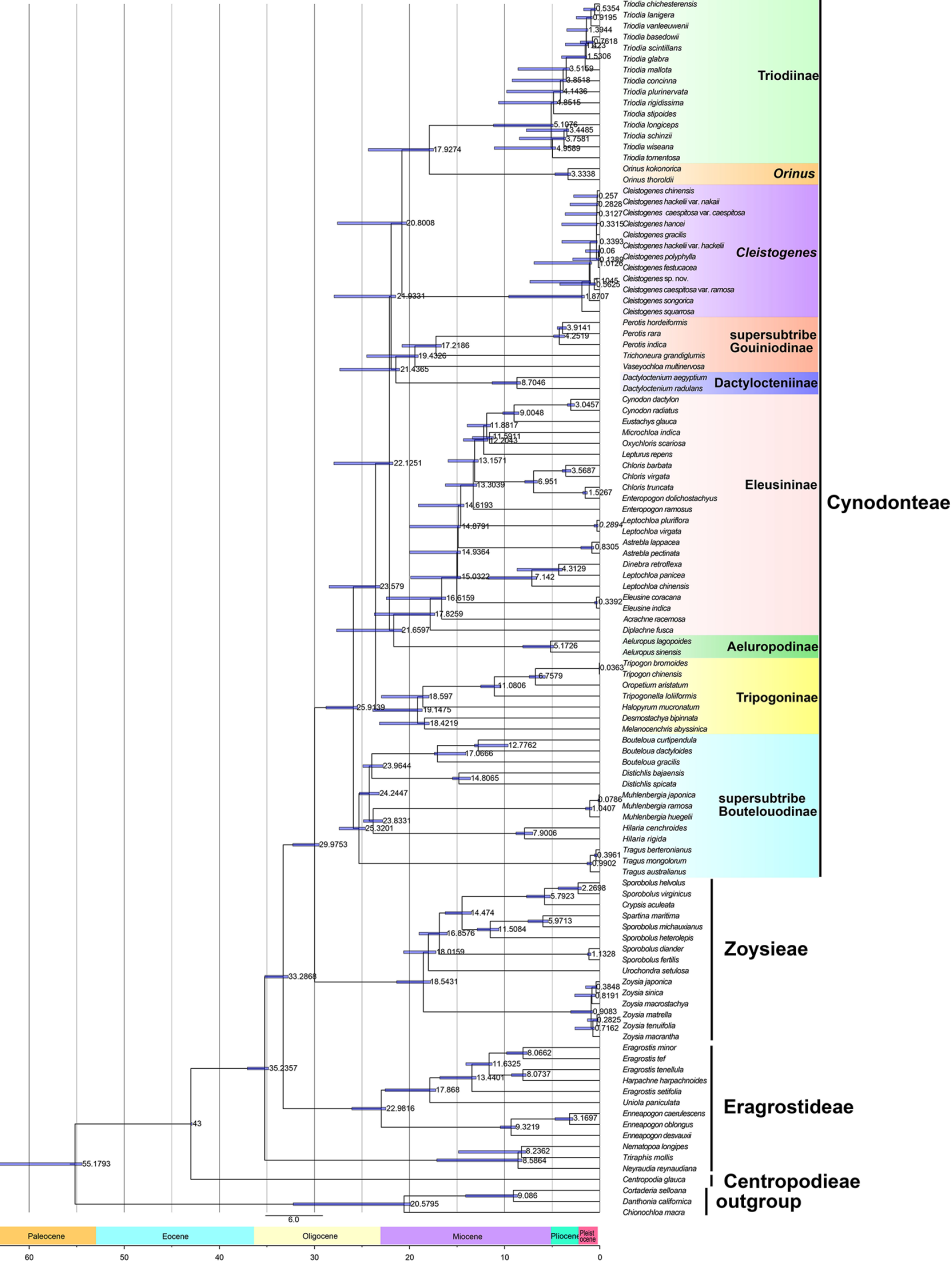
Divergence time estimation of Chloridoideae based on the complete plastome. The numbers next to nodes indicate the estimated median ages of the nodes while the blue bars correspond to the 95% highest posterior density (HPD) of the estimated ages.

The results of divergence time estimations are shown in **Figure 3** and **Table S2**. Chloridoideae was estimated to have originated 55.18 million years ago (54.44-63.35 Ma), Centropodieae at 43 Ma (42.87-43 Ma), Triraphideae at 35.24 Ma (34.83-37.05 Ma), Eragrostideae at 33.29 Ma (32.76-35.29 Ma), Zoysieae at 29.98 Ma (29.49-32.28 Ma), and Cynodonteae at 25.92 Ma (25.45-28.79 Ma). The diversification of tribes was estimated to have begun at 43 Ma (42.87-47-43 Ma), followed by Triraphideae at 8.59 Ma (8.17-17.13 Ma), Eragrostideae at 22.98 Ma (22.49-26.07 Ma), Zoysieae at 18.54 Ma (17.79-21.56 Ma), and Cynodonteae at 25.91 Ma (25.45-28.79 Ma).

Nine lineages of Cynodonteae diverged during the period from the late Oligocene to the early Miocene (20.31-28.79 Within Cynodonteae, supersubtribe Boutelouodinae diverged first (25.91 Ma, 25.45-28.79 Ma). The Tripogoninae stem age was 23.58 Ma (23.08-28.46 Ma) and diverged after supersubtribe Boutelouodinae. The divergence of the Eleusininae-*Aeluropus* clade was estimated around 22.13 Ma (21.76-27.96 Ma). The split between Eleusininae and Aeluropodinae was estimated around 21.66 Ma (20.77-27.67 Ma). Eleusininae began to diversify at 17.83 Ma (17.35-23.7 Ma). The clade composed of supersubtribe Gouiniodinae and Dactylocteniinae diverged at 21.93 Ma (21.46-27.04 Ma). The split between supersubtribe Gouiniodinae and Dactylocteniinae was estimated at 21.44 Ma (21.05-27.33 Ma). The divergence time of the *Cleistogenes*-*Orinus*-*Triodia* clade was estimated at 20.8 Ma (20.31-27.58 Ma). *Cleistogenes* was estimated to have diversified only recently around 1.87 Ma (1.61-9.57 Ma). The *Orinus*-*Triodia* clade was estimated to have diverged at 17.93 Ma (17.49-24.33 Ma).

### Ancestral state reconstructions of Chloridoideae

**Figure 4:**
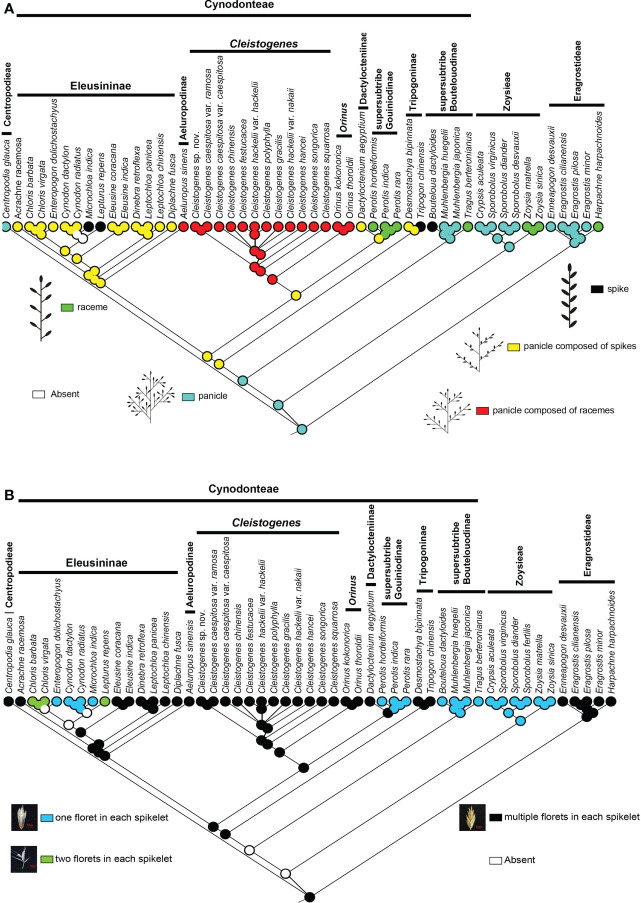
Ancestral state reconstructions of in Chloridoideae: **(A)** inflorescence type; **(B)** spikelet type. The differently coloured spots at the nodes indicate the different character states.

There are five types of interactions in Chloridoideae, including panicle, spike, raceme, panicle composed of spikes, and panicle composed of racemes. The panicle is mainly found in Eragrostideae, Zoysieae, and the early-diverging lineage of Cynodonteae (i.e., supersubtribe Boutelouodinae). Spike and raceme are not common in Chloridoideae. Raceme was only found in *Harpachne*, *Perotis*, *Tragus* and *Zoysia*. Spike was only found in *Bouteloua*, *Lepturus, Microchloa* and *Tripogon*. Panicles composed of racemes were only found in *Aeluropus, Cleistogenes* and *Orinus*, which all have similar aspects of inflorescence type. Panicles composed of spikes existed in Dactylocteniinae, Eleusininae and Tripogoninae. Ancestral state reconstructions showed that the panicle was the ancestral inflorescence state of this group, and the spike, racemes, panicle composed of spikes, and panicle composed of racemes were all derived in Chloridoideae (**Figure 4A**).

According to the number of florets in each spikelet, all the involved taxa can be divided into three types: one floret in each spikelet, two florets in each spikelet, and several florets in each spikelet. Most taxa of Chloridoideae have several florets in each spikelet, and the condition of one floret in each spikelet was observed in Zoysieae (*Crypsis*, *Sporobolus*, and *Zoysia*) and some genera of Cynodonteae (*Bouteloua*, *Muhlenbergia*, *Tragus*, *Perotis*, *Enteropogon*, *Cynodon*, and *Microchloa*). Each spikelet of Chloris and *Lepturus* has two florets. **Figure 4B** shows that several florets in each spikelet are the original state of Chloridoideae, and either one or two florets in each spikelet were derived in Chloridoideae.

**Figure 5:**
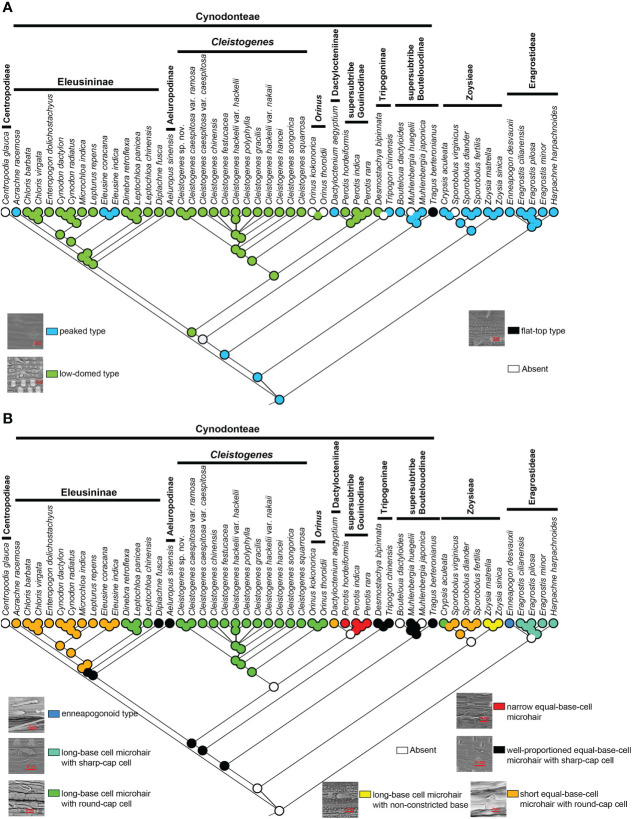
Ancestral state reconstruction of Chloridoideae: **(A)** stomatal subsidiary cell shape; **(B)** microhair type. The differently coloured spots at the nodes indicate the different character states.

Three types of stomatal subsidiary cells were observed in the present study, including the peaked type, low-domed type and flat-top type (**Figure 5B**). The peaked peaked type was mainly found in Eragrostideae, Zoysieae and early-diverging lineages of Cynodonteae (supersubtribe Boutelouodinae). A low-domed type was observed in most Cynodonteae taxa. The flat-top type was only found in *Tragus*. Ancestral state reconstructions showed that the peaked type was the ancestral state, while the low-domed type and the flat-top type are derived (**Figure 5B**).

There are three types of microhairs, including the long-base cell enneapogonoid type, long-base cell chloridoid type and equal-base-cell chloridoid type (**Figure 5B**). Long-base cell chloridoideae include long-base cell microhairs with sharp cap cells, long-base cell microhairs with round cap cells, and long-base cell microhairs with nonconstricted bases. Equal-base-cell microhairs include short equal-base-cell microhairs, relatively narrow equal-base-cell microhairs with round cap cells and well-proportioned equal-base-cell microhairs with sharp cap cells. The morphology of microhairs was diverse in Chloridoideae, and contained significant differences between taxa of the same tribe. In Eragrostideae, microhairs were enneapogonoid type or long-base cell microhairs with sharp cap cells. In Zoysieae, they were long-base cell microhairs with round-cap cells, long-base cell microhairs with nonconstricted bases or short equal-base-cell microhairs with round-cap cells. In Cynodonteae, there was a number of different types of microhairs, including well-proportioned equal-base-cell microhair with sharp-cap cells, unobserved microhairs in supersubtribe Boutelouodinae and Tripogoninae, long-base cell microhair with round-cap cells, short equal-base-cell microhair with round-cap cells or well-proportioned equal-base-cell microhair with sharp-cap cells in Eleusininae, long-base cell microhair with round-cap cells in *Cleistogenes* and *Orinus*, short equal-base-cell microhair with round-cap cells in *Dactylocteinum*, and relatively narrow equal-base-cell microhair cells in *Perotis*. As shown in **Figure 5B**, the ancestral state of the microhair was uncertain.

**Figure 6:**
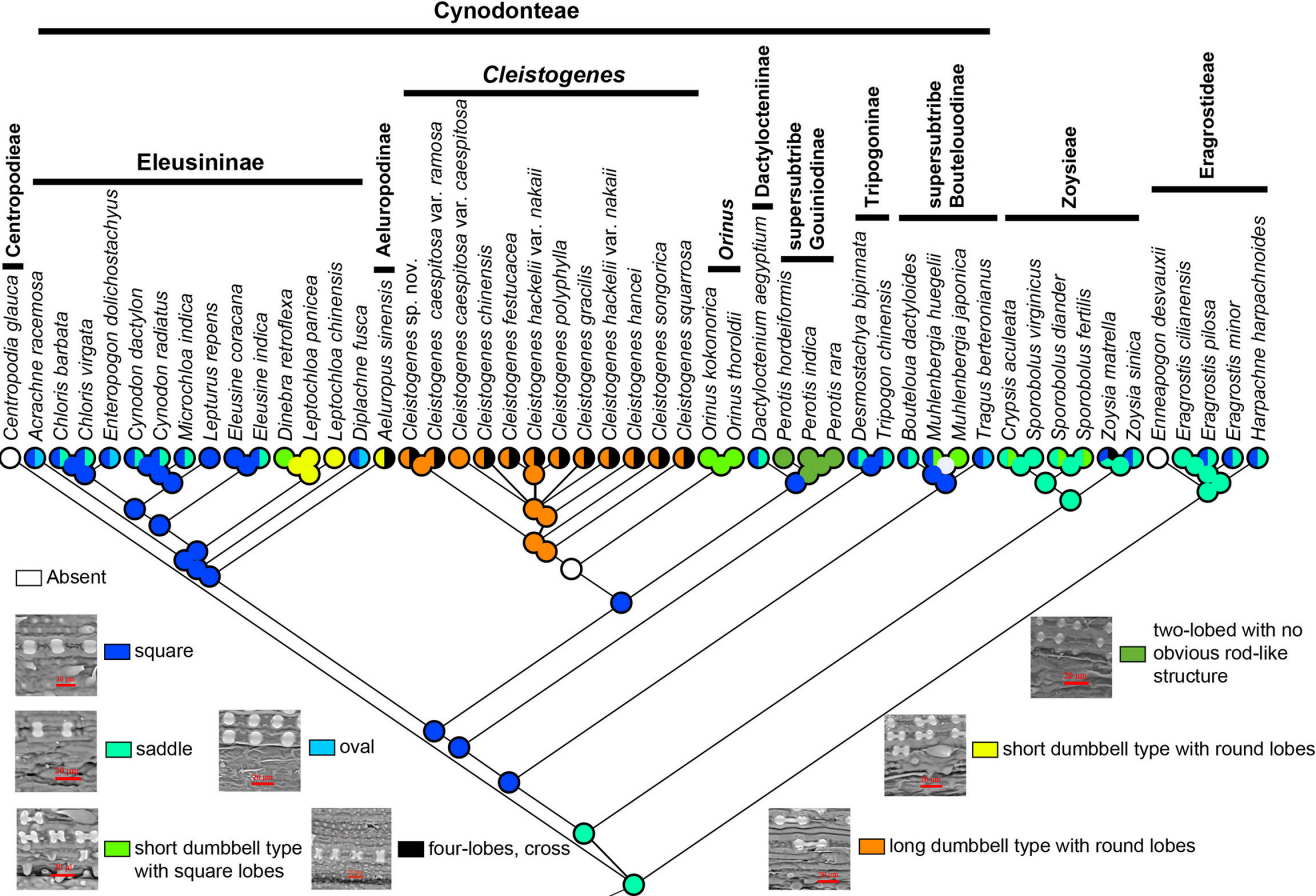
Ancestral state reconstruction of phytoliths in Chloridoideae. The differently coloured spots at the nodes indicate the different character states.

Phytoliths can be classified into four types and nine subtypes in Chloridoideae (**Figure 6**). Four types included mono-lobed, two-lobed with no obvious rod-like structure, two-lobed with obvious rod-like structure and multi-lobed. The mono-lobed type includes square, oval and saddle shapes, and the two-lobes include the short dumbbell type with square lobes, the short dumbbell type with round lobes, and the long dumbbell type with round lobes. The multiple lobes type includes three-lobes type and four-lobes type (cross). The morphology of phytoliths was similar in Eragrostideae and Zoysieae; however, there was a high degree of diversity in Cynodonteae. Square and saddle-shaped phytoliths were observed in Eragrostideae. In Zoysieae, phytoliths were square, saddle, short dumbbell type with square lobes and four-lobes (cross). In Cynodonteae, there were eight types. In supersubtribe Boutelouodinae, there were square and saddle shapes. In Tripogoninae and *Dactyloctenium*, there were two-lobed with no obvious rod-like structure in *Perotis*, long dumbbell types with round lobes and crosses in *Cleistogenes*, and short dumbbell types with square lobes in *Orinus*. Square, oval and saddle types were observed in most taxa of Eleusininae, whereas there were short dumbbell type with round lobes in *Leptochloa*, short dumbbell type with square lobes in *Dinebra*, square and oval in *Diplachne*, and short dumbbell type with round lobes and cross in *Aeluropus*. As shown in **Figure 6**, saddle shapes are original, and other types were derived.

## Discussion

### Features of Chloridoideae plastomes

Plastomes have become useful to the study of phylogenetic relationships in Poaceae. The plastomes of Chloridoideae showed the typical quadripartite structure as previously reported in Poaceae species, e.g., *Oryza sativa* (Hiratsuka et al., 1989), *Zea mays* (Maier et al., 1995), *Brachypodium distachyon* (Bortiri et al., 2008), consisting of an LSC region and an SSC region and separated by a pair of IR regions. They are highly conserved in genome size, structure, GC content, and gene content. Plastid genomes are generally very A+T-rich (Ravi et al., 2008). The GC content of Chloridoideae plastomes is similar to that of other Poaceae plastomes (Wu and Ge, 2012; Liu et al., 2020b). The loss of *rps15* in *Eragrostis tenellula* was previously reported in other studies (Somaratne et al., 2019; Liu et al., 2021a). The position of *rps15* was near the boundary between the IR and SSC regions (Davis and Soreng, 2010). In most species of Poaceae, *trnN*-*GUU* is duplicated in the IR regions, while in *Distichlis spicata*, there was another copy in the LSC region.

### Phylogenetic relationships within Chloridoideae and tribal delimitation

Recent molecular phylogenetic studies showed that Chloridoideae is sister to Danthonioideae (Soreng et al., 2015; Soreng et al., 2017); hence, three Danthonioideae taxa were selected as outgroups in the present study (**Figures 1**, **S1**, **S2**). Chloriodideae was estimated to have originated in the late Palaeocene (55.18 Ma) (**Figure 3**, **Table S2**). There is an abrupt and transient climatic event during the period of Paleocene Eocene thermal maximum (PETM) in the geologic record (Zachos et al., 2001; Zachos et al., 2005). Diversity increased in Poaceae during this period (Jaramillo et al., 2010). The present study provided a robust phylogeny of Chloridoideae (**Figure 1**). In the present study, the general tribal framework within Chloridoideae analyzed was congruent with that inferred from seven plastid regions and ITS sequences (Peterson et al., 2011). Centropodieae diverged in Eocene (42.87-43 Ma), while the other three tribes of Chloridoideae diverged in the Oligocene (25.45-37.05 Ma) (**Figure 3**, **Table S2**). In the clade composed of Eragrostideae, Zoysieae and Cynodonteae, the evolution of C_4_-specific isoform of phosphoenol-pyruvate carboxylase (PEPC) was through a gene duplication of a non-C_4_ PEPC gene followed by neofunctionalization (Christin et al., 2007). The duplication that occurred in this clade evolved a C_4_-specific PEPC independently. The diversification of these three tribes may be caused by the CO_2_ decline in the Oligocene (Christin et al., 2008).

### Phylogenetic relationships among subtribes and genera of Cynodonteae

The phylogenomic framework within Cynodonteae was incongruent with previous studies (Peterson et al., 2010; Soreng et al., 2015; Peterson et al., 2016; Soreng et al., 2017). The phylogeny of this tribe circumscribed nine monophyletic groups with strong support, which coincided with previous phylogenetic studies based on small-scale plastid datasets and nuclear datasets (Peterson et al., 2010; Peterson et al., 2011; Duvall et al., 2016). There were some differences in the relationship between the nine groups. Tripogoninae and Aeluropodinae obtained remarkably different placements from some previous studies. Tripogoninae was sister to supersubtribe Boutelouodinae in most studies (Peterson et al., 2010; Soreng et al., 2015; Peterson et al., 2016; Soreng et al., 2017). Aeluropodinae was sister to Triodiinae, Orininae or a clade comprising *Orinus* and Triodiinae in previous studies, while it was sister to Eleusininae in the present study (**Figures 1**, **S1**). Many internal phylogenetic relationships of Cynodonteae were resolved with strong support except for the clade composed of *Cleistogenes*, *Orinus* and Triodiinae. Orininae (including *Cleistogenes* and *Orinus*) was proposed based on the sister relationship between these two genera with moderate support. The clade comprising *Cleistogenes*, *Orinus* and Triodiinae is weakly supported (**Figure 1**).

The incongruences of phylogenetic relationships within Cynodonteae may be caused by the rapid diversification during a short time period. Cynodonteae was estimated to have experienced rapid divergence within a short time period (**Figure 3**), which could be a major obstacle in resolving phylogenetic relationships within Cynodonteae. Atmospheric CO_2_ concentrations decreased significantly during the Oligocene and reached modern levels in the late Oligocene (Pagani et al., 2005). C_4_ evolution has been seen as a result of a decline of atmospheric CO_2_ concentrations (Sage, 2001; Osborne and Beerling, 2006; Christin et al., 2008). Diversification within Chloridoideae was especially active during the period of the Late Oligocene to the Pleistocene (**Figure 3**), which coincides with the expansion of grasses that began in the Oligocene (Christin et al., 2008). A significant climate transition occurred in the Oligocene/Miocene boundary. Ocean temperatures slowly increased, and continental ice volume apparently decreased in the period of the middle to late Oligocene (Stott et al., 1990; Miller et al., 1991). It is significant that there is an adaptive transition from a wetter and shaded environment to a drier open habitat occurred in Chloridoideae (Bouchenak-Khelladi et al., 2010). These conditions may have caused Cynodonteae to diversify during this period.

The basal lineage of Cynodonteae in the plastome-based phylogenetic tree is the supersubtribe Boutelouodinae, which includes *Bouteloua*, *Distichlis*, *Muhlenbergia*, *Hilaria* and *Tragus* (**Figures 1**, **S1**, **S2**). Supersubtribe Boutelouodinae was newly established in 2017 (Soreng et al., 2017) following the worldwide phylogenetic classification of Poaceae in 2015 (Soreng et al., 2015), and consists of six subtribes, including Boutelouinae, Hilariinae, Monanthochloinae, Muhlenbergiinae, Scleropogoninae and Traginae. Recent phylogenetic studies showed that supersubtribe Boutelouodinae was positioned sister to Tripogoninae with a low bootstrap value (Peterson et al., 2010; Peterson et al., 2012; Soreng et al., 2015; Soreng et al., 2017), strongly supporting these two groups as the successive early-diverging groups of Cynodonteae in the present study (**Figures 1**, **S1**). The discrepancy between previous studies and the present study was generally attributed to the rise of phylogenetic information. This discrepancy has a wide scope in terms of the distribution of this supersubtribe. Genera of Boutelouinae and Scleropogoninae were distributed in South and North America, genera of Monanthochloinae (*Distichlis*) were distributed in South America, North America and Australia, and genera of Muhlenbergiinae (*Muhlenbergia*) were distributed in South America, North America and Asia. Subtribe Traginae is distributed in Africa, and as a genus of this subtribe, *Tragus*, has a wide distribution in Africa, Asia, Europe and Australia. Different types of dicliny occur in these groups and are restricted to the western hemisphere in Chloridoideae (Beauvois, 1812). Ancestral state reconstructions of five characters showed that the root of Chloridoideae is panicle, with several florets in each spikelet, peaked-type stomatal subsidiary cells, and saddle-shaped phytoliths (**Figures 4A, B**, **5A, B**, **6**). These morphological characteristics changed during the process of species generation and migration and were generated to adapt to new habitats. Previous studies have shown the diversity of inflorescences and spikelets, particularly in Cynodonteae (Liu et al., 2005; Liu et al., 2007; Muchut et al., 2017; Muchut et al., 2019; Pilatti et al., 2019). As the basal lineage of Cynodonteae, species of supersubtribe Boutelouodinae have similar types of inflorescences, spikelets and stomatal subsidiary cells as Eragrotideae and Zoysieae species (**Figures 4A, B**, **5**). The time tree shows that supersubtribe Boutelouodinae split from Cynodonteae and returned to the late Oligocene (**Figure 3**).

The phylogenetic position of Aeluropodinae in Cynodonteae was uncertain in previous phylogenetic studies. The position of Aeluropodinae differed in various tree analyses, it was sister to *Triodia* in the ITS tree and sister to the clade composed of Triodiinae and *Orinus* in the plastid tree and combined tree, however, these relationships all had low bootstrap values and moderate posterior probability values (Peterson et al., 2010). In the present study, Aeluropodinae obtained a remarkably different placement compared with previous studies (**Figures 1**, **S1**). Plastome data strongly supported that Aeluropodinae was sister to Eleusininae (**Figures 1**, **S1**). Aeluropodinae is a subtribe of Eurasian and African plants in the grass family, found primarily in desert regions (Clayton et al., 2008). *Aeluropus* diverged in the early Miocene (**Figure 3**). During the Eocene-Oligoceae period, the elevation of the Tibetan Plateau resulted in increased drought in Central Asia (Zhang et al., 2018; Clift and Webb, 2019). The divergence of this genus may be related to the arid environment. The decrease in soil moisture caused by drought can lead to an increase in soil salinity. Species of Aeluropodinae are distributed in saline environments. *Aeluropus lagopoides* and *Aeluropus littoralis* can be used as fodder and forage grass and to study salt tolerance (Ahmed et al., 2013; Azri et al., 2016; Zamin et al., 2019; Younesi-Melerdi et al., 2020). *Aeluropus lagopoides* can survive and reproduce in highly saline inland and coastal conditions under warm temperatures (Gulzar and Khan, 2001). It seems that the proximate strategy of *Aeluropus lagopoides* is to use vegetative methods for the recruitment of new individuals as a less costly way of recruitment in a highly unpredictable, harsh environment. Plasome data obtained remarkably different placements of Aeluropodinae compared with previous studies (**Figures 1**, **S1**). The morphology study showed that Aeluropodinae has similarities with both the *Cleistogenes*-*Orinus* clade and Eleusininae in spikelet, stomatal subsidiary cell and phytolith (**Figures 4B**, **5A**, **6**); however, the morphology of microhairs is different from both of them (**Figure 5B**). Microhair exists in most taxa of Poaceae, which have a basal and a distal cell (Watson et al., 1985). In some grasses of the subfamily Chloridoideae, microhairs act as “salt glands” that can secrete excess salts to the environment (Warren and Brockelman, 1989; Marcum et al., 1998; Marcum and Pessarakli, 2006; Oi et al., 2014). *Aeluropus* species are typical halophytic grasses, and the morphology of microhairs is closely related to their functions (Liu et al., 2006; Barhoumi et al., 2008).

Orininae (including *Cleistogenes* and *Orinus*) was recognized as a new subtribe of Cynodonteae based on the moderately supported sister relationship in a combined tree of six plastid and ITS sequences (Peterson et al., 2016). In previous studies, *Cleistogenes* and *Orinus* were classified in Cynodonteae, and were not classified at the subtribe level (Peterson et al., 2010; Soreng et al., 2015). The morphological differences between these two genera are lemma and underground part. Hairy lemma and long scaly rhizome were observed in *Orinus* but not in *Cleistogenes*. Comparative plastomes and plastome phylogenomic analysis of these three genera were performed, and the results showed a similar pattern in plastome structure, gene order, gene content, IR boundaries, the type and number of repeat sequences and codon usage (Wang et al., 2021). In the present study, *Orinus* is sister to *Triodia*, while *Cleistogenes* was sister to the clade comprising *Orinus* and *Triodia* (**Figures 1**, **S1**, **S2**). The same results were previously published, and plastome data did not support the establishment of Orininae (Wang et al., 2021).

The phylogenetic position of *Acrachne* has been controversial for a long time. *Acrachne* is either classified under Dactylocteniinae or Eleusininae due to its different phylogenetic positions in the ITS tree and plastid tree (Peterson et al., 2010; Peterson et al., 2015; Soreng et al., 2015; Peterson et al., 2016; Soreng et al., 2017). The phylogenetic position of *Acrachne* as determined with plastid-based phyogenetic analysis was within Eleusininae; however, it was sister to *Dactyloctenium* in the Dactylocteniinae ITS tree (Peterson et al., 2015; Peterson et al., 2016). *Acrachne*, *Dactyloctenium* and *Eleusine* have close phylogenetic relationships and form a distinctive cluster in a numerical analysis (Phillips, 1982). These three genera have common characteristics, such as the secund spikes of overlapping spikelets and the peculiar ornate grain within a free pericarp; these characteristics are not usual features in Poaceae (Phillips, 1982). *Acrachne* has a similar distribution to Dactylocteniinae and Eleusininae. Its native range is tropical and subtropical in the old world to North Australia. Some *Acrachne* taxa were placed in *Eleusine*. The rugose grain with a caducous pericarp had a character similar to that of these two genera, while there were up to 20 florets in each spikelet, and lemma keel was produced into a mucro or cusp in *Acrachne*. *Eleusine multiflora* was recognized as a link between *Eleusine* and *Acrachne* (Phillips, 1972). Ahmad et al. (Hubbard, 1936) reported that *Acrachne racemosa* is identified by dumb bell-shaped or cross-shaped phytoliths on leaf epidermis, while saddle-shaped phytoliths are present in other Eragrostideae taxa. Liu et al. (2010) found that *Acrachne*, *Dactyloctenium* and *Eleusine* have similar long cell outling, bicellular microhairs and macrohair micromorphological characters of lemma. Soreng et al. (2017) considered that there was a possible hybridization event and subsequent genomic introgression between *Acrachne racemosa* (B. Heyne ex Roem. & Schult.) Ohwi and an unknown member of Eleusininae since the incongruence between phylogenetic relationships based on plastid and nuclear DNA markers.

*Acrachne* is the second diverged group in Eleusininae based our plastome data, and should be classified into Eleusininae (**Figures 1**, **S1**, **S2**). In the analysis of ancestral state reconstruction, *Acrachne* has similarities with Eleusininae and *Dactyloctenium* in inflorescence, spikelet, stomatal subsidiary cells, microhair and phytolith morphology (**Figures 4**–**6**). These results indicated that these three groups may have close phylogenetic relationships. and is consistent with previous opinions (Phillips, 1982). *Diplachne* and *Leptochloa* were both established by Beauvois (1812). Kunth (1815) placed *Diplachne* and *Leptochloa* in different tribes. Meisner (1843) placed *Diplachne* adjacent to *Leptochloa* in Chlorideae. *Diplachne* and *Leptochloa* were once recognized as closely related genera of Eragrosteae (McNeill, 1979). It was not clear that whether *Diplachne* could be recognized as a distinct genus. Many researchers included *Diplachne* in *Leptochloa* for no valid reason (Hubbard, 1936). Analysis of morphological and anatomical data demonstrated the polyphyly of *Leptochloa* (including *Diplachne*) (Snow, 1998). It was found that *Diplachne* and *Leptochloa* overlapped in a numerical analysis of morphological characters (Phillips, 1982). Morphologically, *Diplachne* and *Leptochloa* can be distinguished from each other, but they are similar in inflorescence structure. Lemmas of *Diplachne* are shortly awned, whereas those of *Leptochloa* are awnless. In recent molecular phylogenetic studies, it was found that there are three and five strongly supported separate lineages of *Leptochloa* in phylogenetic trees based on seven and six DNA markers, respectively. Among the five lineages, one lineage corresponds to *Diplachne*, which is located between *Dinebra* and *Leptochloa* (Peterson et al., 2012), which are widely distributed in warmer temperate regions. The present study showed that *Diplachne* and *Leptochloa* are in different positions of Eleusininae (**Figures 1**, **S1**, **S2**). Plastome data supported these two genera as two distinct genera. *Diplachne* and *Leptochloa* were similar in the morphology of inflorescence, spikelet and stomatal subsidiary cells (**Figures 4A, B**, **5A**), while they were noticeably different in morphology of microhair and phytolith (**Figures 5B**, **6**). Microhairs have been detected on leaf epidermis and function as salt glands in Chloridoideae (Amarasinghe and Watson, 1989; Kobayashi et al., 2007). Grass phytoliths are very diverse and show a high degree of multiplicity (Rovner, 1971). The different morphologies of microhairs and phytoliths may be related to the different ecological habitats occupied by these two genera.

### Morphological character evolution

#### Inflorescence

Grass inflorescences and spikelets are diverse, developmentally complex, and can be used to distinguish grass species (Malcomber et al., 2006). Inflorescences have played important roles in systematic and phylogenetic studies (Kirchoff and Claßen-Bockhoff, 2013). They act as functional units in plant reproduction and are largely shaped by natural selection. Previous studies displayed a fascinating inflorescence and spikelet diversity in Chloridoideae (Clayton, 1982; Peterson et al., 1997). The evolution of inflorescence in Poaceae is assumed to be random because of the diversity (Muchut et al., 2019). The ancestral state of Chloridoideae is panicle, and the raceme and spike forms have independently evolved in some nonsister tribes (**Figure 4A**). Muchut et al. (2019) showed that the free transition among character states was a frequent evolutionary event in Poaceae. Inflorescence and spikelet type have close relationships with the timing and position of pollen presentation, the timing of seed maturation, the extent of seed provisioning, and the extent of seed dormancy (Prieto-Baena et al., 2003). The diversity of inflorescence types probably represents adaptive responses to changes in environment (Harder and Johnson, 2009; Harder and Prusinkiewicz, 2012). The MRCA (most recent common ancestor) of Chloridoideae has panicle inflorescence and the inflorescence types of the MRCA of Eragrostideae, Zoysieae and Cynodonteae are panicle (**Figure 4A**). There are two transition points of inflorescence type in Cynodonteae. The inflorescence type of the MRCA of Cynodonteae taxa, excluding supersubtrbe Boutelouodina, is panicle composed of spikes (23.08-28.46 Ma). The inflorescence type of the MRCA of *Cleistogenes* and *Orinus* is panicle composed of racemes (20.31-27.58 Ma). These two points occur in the period of late Oligocene to early Miocene (**Figure 3**). The panicle is the most common inflorescence in Poaceae. Inflorescences were inferred to have evolved from simple to complex (Pilatti et al., 2018). In the present study, the finding was the opposite. This finding indicated that the evolution of inflorescences may be a process that tends to be oversimplified.

#### Spikelet

Spikelet is also an important evolutionary unit in grass inflorescences. Grass inflorescences are built of repeated units called spikelets, which consist of a pair of glumes (bracts) enclosing a cluster of one to as many as 40 flowers, the number of which depends on the species. The spikelet is the specific unit of the grass inflorescences. The MRCA of Chloridoideae and Eragrostideae has multiple florets in each spikelet (**Figure 4B**). A spikelet of the MRCA of Zoysieae has one floret (17.79-21.36 Ma) (**Figure 3**). A transition point of spikelet type occurred in Chloridoideae in the period of Miocene (**Figures 3**, **4A**). In Miocene, the climate transition may cause the number of seeds that can germinate and grow normally reduced. The increase in floret number increases the number and survival rate of seeds.

#### Shape of stomatal subsidiary cell

In Poaceae, a stomatal complexe consists of two elongated and dumbbell-shaped guard cells (GCs)and two closely associated lateral subsidiary cells (SCs). It has been recognized that the composition and morphology of stomata are closely associated with fast stomatal responses in Poaceae (Stebbins and Shah, 1960; Franks and Farquhar, 2007). The four-celled stomatal complex of Poaceae is innovative and allows for larger pore apertures and faster responsiveness to environmental changes than other stomatal types (Nunes et al., 2020). GCs are the centers of stomatal complexes, which surround and regulate the size of the stomatal pore. The shapes of SCs and GCs were recognized to play significant roles in improving stomatal kinetics (Stebbins and Shah, 1960; Franks and Farquhar, 2007). The different types of stomatal subsidiary cells in Chloridoideae may be related to environmental adaptation. The diversification of stomatal subsidiary cells mainly occurs in Cynodonteae (**Figure 5A**). The MRCA of Chloridoideae is peaked type stomatal subsidiary cells. The stomatal subsidiary cells of the MRCA of Eragrostideae, Zoysieae and Cynodonteae are peaked type. There are multiple transition points of stomatal subsidiary cells in Chloridoideae. The stomatal subsidiary cell of the MRCA of Cynodonteae taxa, excluding supersubtrbe Boutelouodinae and Tripogoninae, is the low-domed type (21.76-27.96 Ma), which evolved in the late Oligocene to the early Miocene (**Figures 3**, **5A**). The diversity of stomatal subsidiary cell shape may have been an adaptation to lower CO_2_ concentrations during the Paleogene (Pagani et al., 2005).

#### Microhair

In Poaceae, a microhair often consists of a pair of cells, microhairs are hence called bicellular microhairs. It was found that microhairs have been detected in many grass species (Liphschitz and Waisel, 1982; Kobayashi, 2008), but they only function as salt glands in Chloridoideae (Amarasinghe and Watson, 1989; Céccoli et al., 2015). An unusual arrangement of plasma membranes called ‘partitioning membranes’ was observed in ultrastructural studies of microhairs and showed that they were involved in the process of salt secretion (Oross and Thomson, 1982b; Oross and Thomson, 1982a). In the present study, different shapes of basal cells and cap cells of microhair were found in Chloridoideae, while ‘partitioning membranes’ were not observed (**Figure 5B**). With the exception of the enneapogonoid type, all the other types are chloridoid type microhairs. Chloridoid type microhairs can also be divided into different types according to the shape and relative length of basal cell and cap cell. The microhairs of the MRCA of Chloridoideae, Eragrostideae and Zoysieae are unknown (**Figure 5B**). The MRCA of Cynodonteae has a well-proportioned equal-base-cell microhair with sharp-cap cells (25.45-28.79 Ma) (**Figures 3**, **5B**). The MRCA of Eleusininae, excluding *Diplachne*, has a short equal-base-cell microhair with round-cap cells (17.35-23.7 Ma) (**Figures 3**, **5B**). The MRCA of *Cleistogenes* and *Orinus* has a long-base cell microhair with round-cap cells (20.31-27.58 Ma) (**Figures 3**, **5B**). The microhair type of Chloridoideae transitioned at three time points in the Oligocene and Miocene periods, which might correspond to the adaptive transition from a wetter and shaded environment to a drier open habitat (Bouchenak-Khelladi et al., 2010).

#### Shape of phytolith

Silica is absorbed through roots and deposited within intercellular spaces and inside the cells of numerous plants resulting in phytoliths or silica bodies (Blackman and Parry, 1968). Previous studies have demonstrated the morphological diversity of phytoliths in Chloridoideae (Twiss et al., 1969). The morphology of phytoliths may be related to habitat, with the most recently differentiated groups preferring open habitats, while the earliest differentiated groups prefer closed habitats (Palmer and Tucker, 1986). Long dumbbell-shaped and short dumbbell-shaped phytoliths were different in the length of the shank, which connected two lobes. It was proposed that the length of the shank strongly related to water availability rather than to grass phylogeny by discriminating morphological types of phytoliths (Barboni and Bremond, 2009). The chemical integrity of phytoliths is also determined by size and shape. The phytoliths of the MRCA of Chloridoideae, Eragrostideae and Zoysieae are saddle shaped (**Figure 6**). **Figure 3** and **Figure 6** showed that the MRCA of Cynodonteae has square-shaped phytoliths (25.45-28.79 Ma). The MRCA of *Cleistogenes* has a long dumbbell type with round lobes (1.61-9.57 Ma). There are two transition points of the phytoliths shape that occur in Chloridoideae. The first point occurs in the Oligocene, representing the square shape transforming into a saddle shape. These two types are very similar and may be evolutionarily related. Poaceae is a phytolith-rich group, the transition of phytoliths type in Chloridoideae coincides with the time of grasslands expansion. It was associated with CO_2_ decline in Oligocene (Christin et al., 2008). The diversification of phytolith-rich plants may be caused by the evolution of large mammalian grazers possessing abrasion-adapted dentition (Beerling and Osborne, 2006; Strömberg, 2011). The second point occurs during the period from the late Miocene to the Pleistocene, when dumbbell-shaped phytoliths occurred in *Cleistogenes* and *Orinus* (**Figures 3**, **6**). Dumbbell-shaped phytoliths were observed to occur largely in C_4_ grasses that flourish in warm, tropical to subtropical regions with a moderate amount of available soil moisture (Twiss, 1992). The occurrence of dumbbell-shaped phytoliths was considered to be strongly linked to environmental factors.

## Conclusions

Phylogenetic analyses showed that Chloridoideae is a well-supported monophyletic group. A total of nine monophyletic lineages were revealed in Cynodonteae: supersubtribe Boutelouodinae, Tripogoninae, Aeluropodinae, Eleusininae, Dactylocteniinae, supersubtribe Gouiniodinae, *Cleistogenes*, *Orinus*, and Triodiinae. Cynodonteae was estimated to have experienced rapid divergence within a short period of time, which could be a major obstacle in resolving the phylogenetic relationships within Cynodonteae. The results of ancestral character state reconstructions demonstrated that the MRCA of Chloridoideae has a panicle, multiple florets in each spikelet, a peaked type of stomatal subsidiary cells, and saddle-shaped phytoliths. Phylogenetic analyses, divergence time estimations and ancestral character state reconstructions provide new insights into the phylogenetic relationships and character evolution of Chloridoideae. Some issues remain to be addressed, and more taxon and additional variable molecular markers are needed for further study.

## Data availability statement

The data presented in the study are deposited in GenBank (https://www.ncbi.nlm.nih.gov/genbank/), accession numbers MW194080-MW194094, OM307668-OM307692.

## Author contributions

XJ-Q, S-JF and X-JZ designed the experiments. All authors took part in the fieldwork. RW carried out the experiment and analyzed the data. RW wrote the first draft of the manuscript. All authors revised and approved the final manuscript. All authors contributed to the article and approved the submitted version.

## Funding

This work was supported by the National Natural Science Foundation of China (31170173 and 31470298) and the survey of herbaceous plant germplasm resources of Shandong Province (2021001).

## Conflict of interest

The authors declare that the research was conducted in the absence of any commercial or financial relationships that could be construed as a potential conflict of interest.

## Publisher’s note

All claims expressed in this article are solely those of the authors and do not necessarily represent those of their affiliated organizations, or those of the publisher, the editors and the reviewers. Any product that may be evaluated in this article, or claim that may be made by its manufacturer, is not guaranteed or endorsed by the publisher.

## Supplementary material

Supplementary Figure 1 Phylogenetic relationships of Chloridoideae inferred from maximum likelihood (ML) and Bayesian inference (BI) based on protein-coding genes. Support values marked above the branches follow the order bootstrap value (BS)/posterior probability (PP).

Supplementary Figure 2 Phylogenetic relationships of Chloridoideae inferred from maximum likelihood (ML) and Bayesian inference (BI) based on noncoding regions. Support values marked above the branches follow the order bootstrap value (BS)/posterior probability (PP).

Supplementary Figure 3 Simplified diagrams of inflorescences found in Chloridoideae. **(A)** raceme, **(B)** spike, **(C)** panicle composed of spikes, **(D)** panicle, **(E)** panicle composed of racemes.

Supplementary Figure 4 Spikelet type found in Chloridoideae. **(A)** one floret in each spikelet, **(B)** two florets in each spikelet, **(C)** multiple florets in each spikelet. Red scale bars: 1 mm.

Supplementary Figure 5 Stomatal subsidiary cell shape found in Chloridoideae. **(A)** flat-top type, **(B)**peaked type, **(C)** low-domed type. Red scale bars: 20 μm.

Supplementary Figure 6 Microhair type found in Chloridoideae. **(A)** enneapogonoid type, **(B)** long-base cell microhair with sharp-cap cell, **(C)** long-base cell microhair with round-cap cell, **(D)** long-base cell microhair with non-constricted base, **(E)**narrow equal-base-cell microhair, **(F)**well-proportioned equal-base-cell microhair with sharp-cap cell, **(G)**short equal-base-cell microhair with round-cap cell. Red scale bars: 20 μm.

Supplementary Figure 7 Phytoliths found in Chloridoideae. **(A)** square, **(B)** saddle, **(C)** oval, **(D)** short dumbbell type with square lobes, **(E)** four-lobes, cross, **(F)** two-lobed with no obvious rod-like structure, **(G)** short dumbbell type with round lobes, **(H)** long dumbbell type with round lobes. Red scale bars: 20 μm.
