# Supplementary figures and images for: Plastid phylogenomics and morphological character evolution of Chloridoideae (Poaceae)
Source: Front Plant Sci. 2022 Nov 2;13:1002724. doi: 10.3389/fpls.2022.1002724 (PMC9666777; doi:10.3389/fpls.2022.1002724)

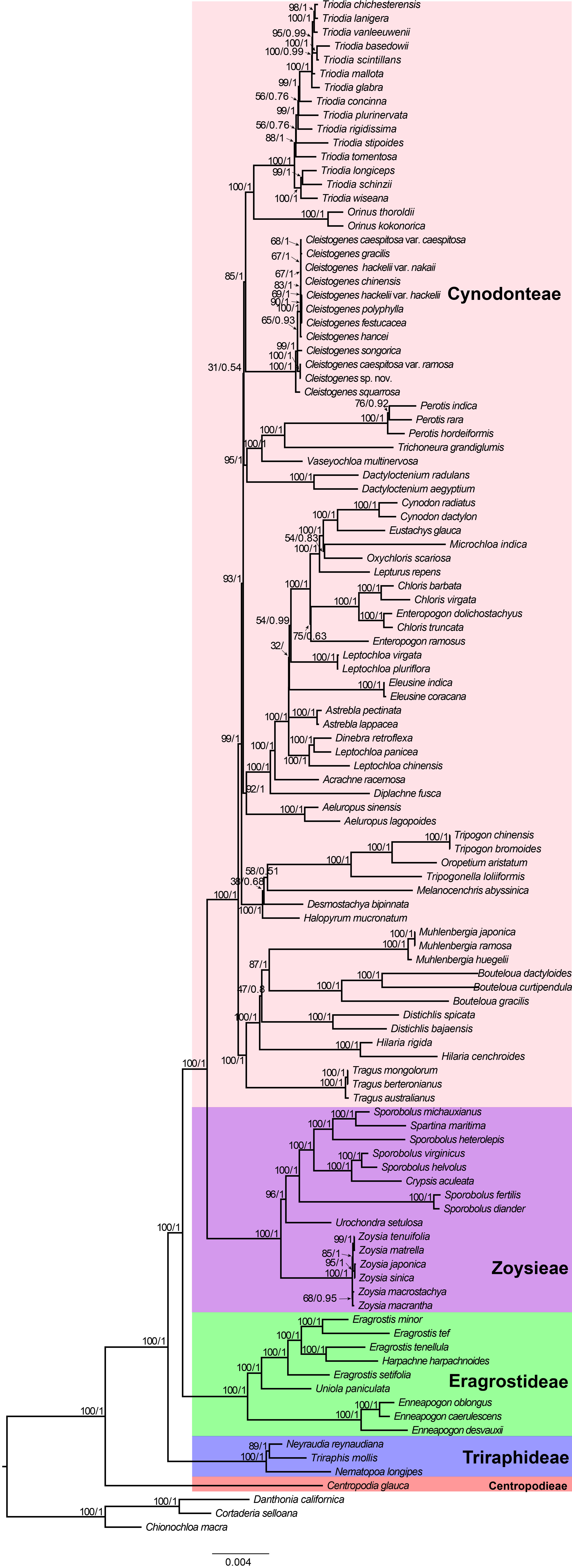

Supplement: Supplementary Figure 1 — Phylogenetic relationships of Chloridoideae inferred from maximum likelihood (ML) and Bayesian inference (BI) based on protein-coding genes. Support values marked above the branches follow the order bootstrap value (BS)/posterior probability (PP). [file Image_1.jpeg]

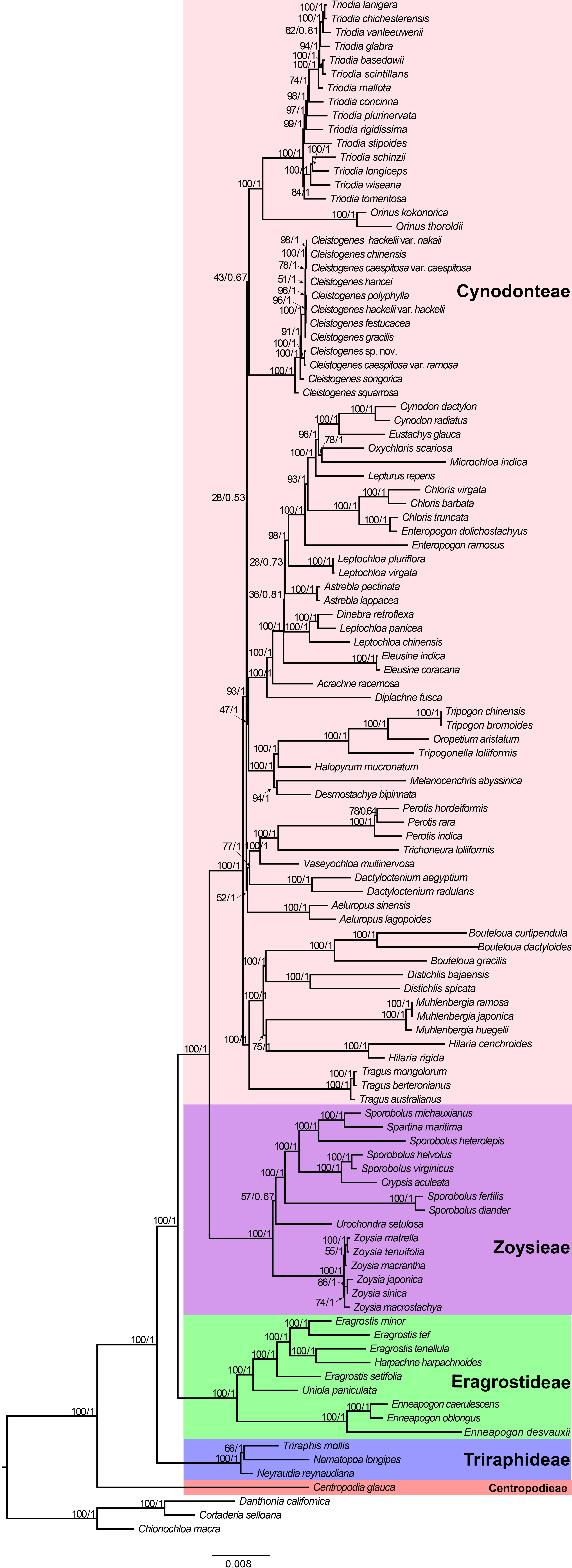

Supplement: Supplementary Figure 2 — Phylogenetic relationships of Chloridoideae inferred from maximum likelihood (ML) and Bayesian inference (BI) based on noncoding regions. Support values marked above the branches follow the order bootstrap value (BS)/posterior probability (PP). [file Image_2.jpeg]

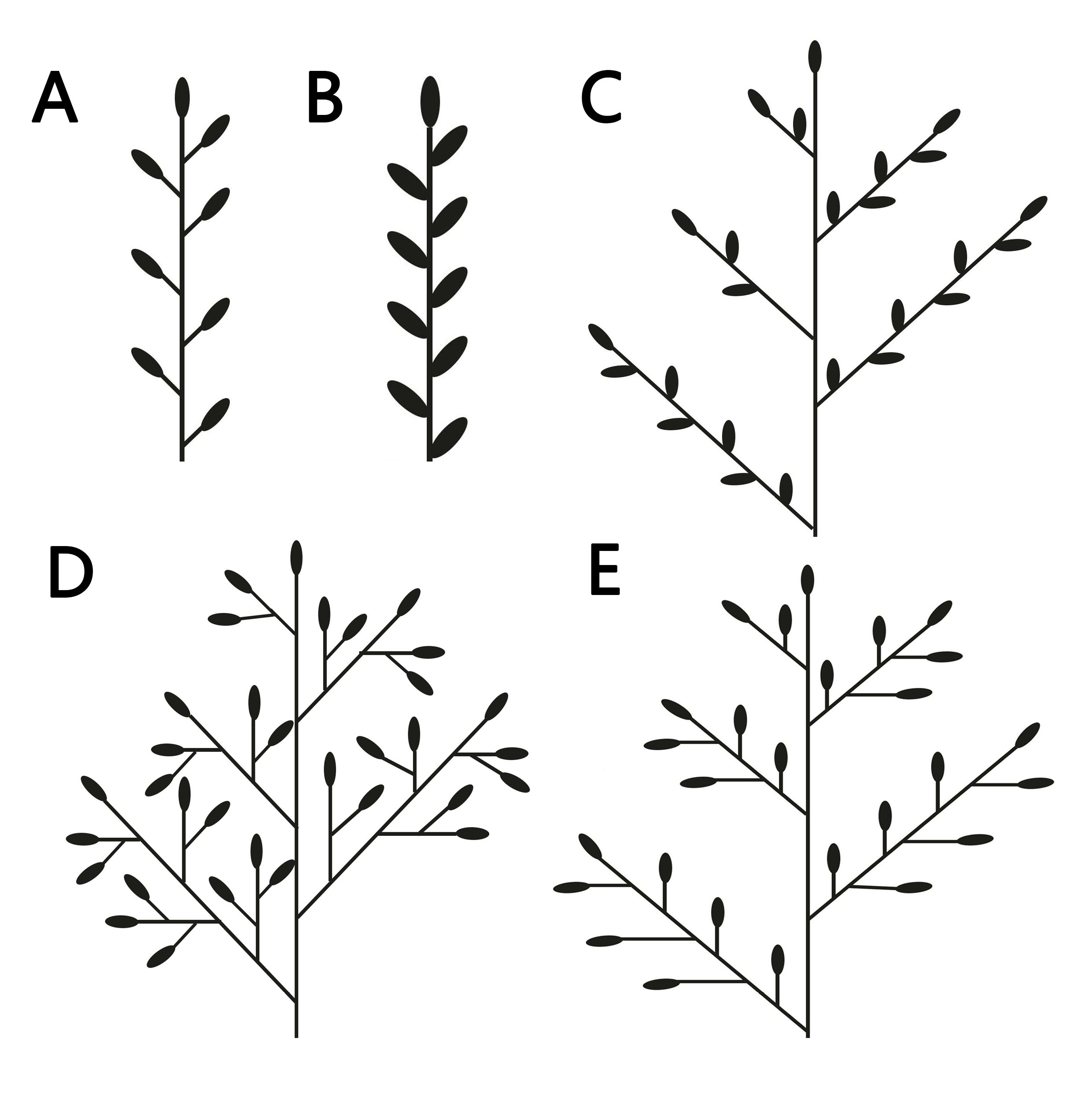

Supplement: Supplementary Figure 3 — Simplified diagrams of inflorescences found in Chloridoideae. (A) raceme, (B) spike, (C) panicle composed of spikes, (D) panicle, (E) panicle composed of racemes. [file Image_3.jpeg]

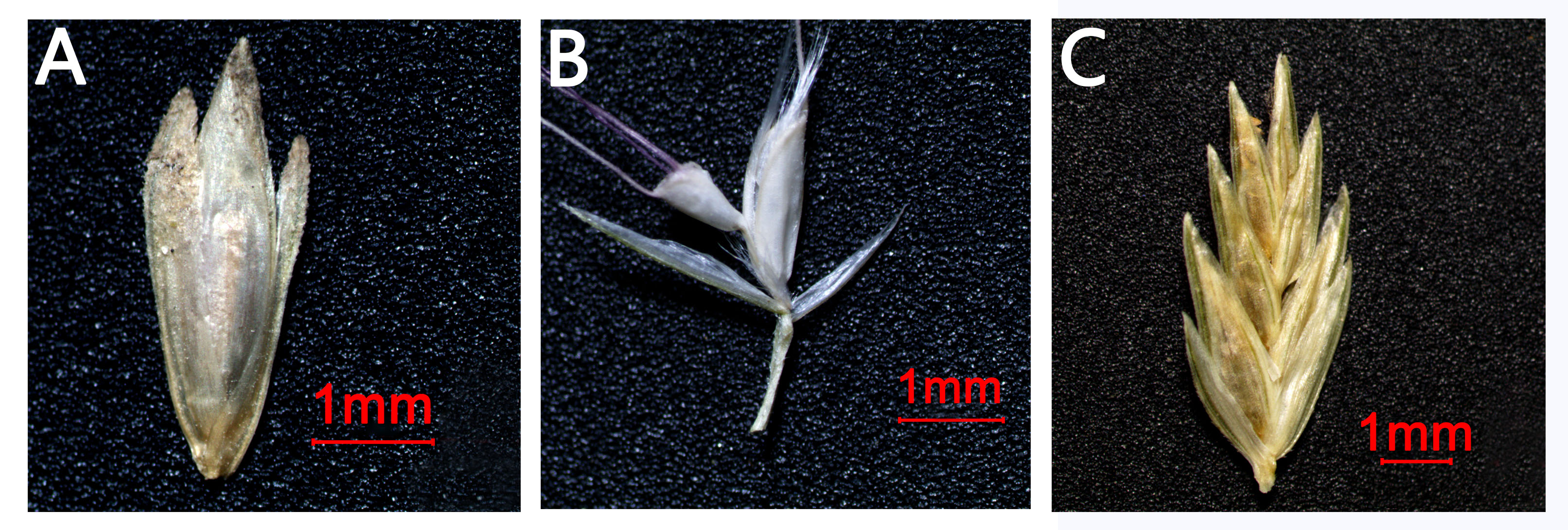

Supplement: Supplementary Figure 4 — Spikelet type found in Chloridoideae. (A) one floret in each spikelet, (B) two florets in each spikelet, (C) multiple florets in each spikelet. Red scale bars: 1 mm. [file Image_4.jpeg]

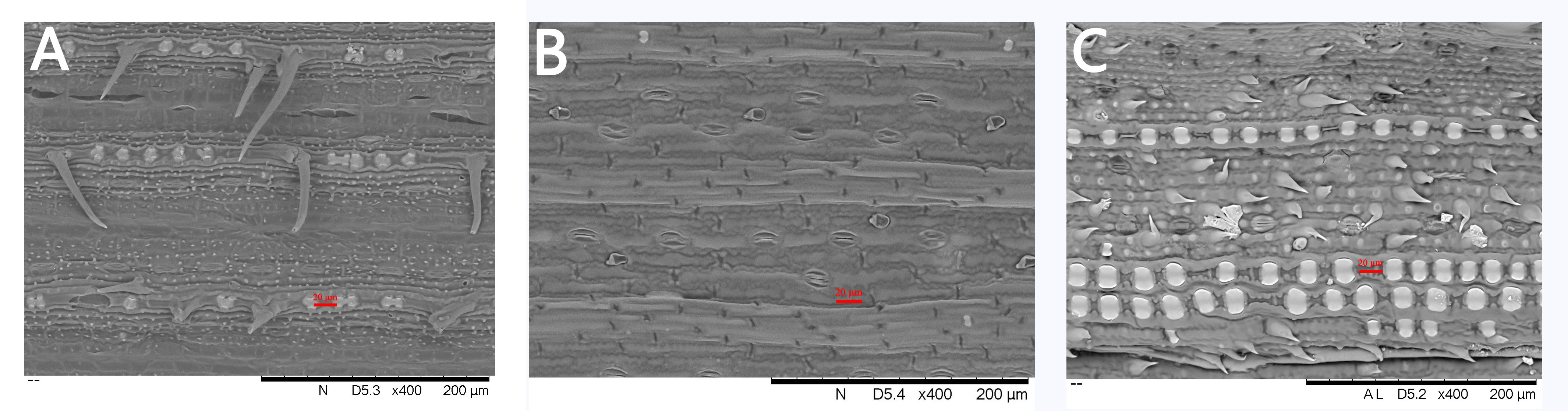

Supplement: Supplementary Figure 5 — Stomatal subsidiary cell shape found in Chloridoideae. (A) flat-top type, (B)peaked type, (C) low-domed type. Red scale bars: 20 μm. [file Image_5.jpeg]

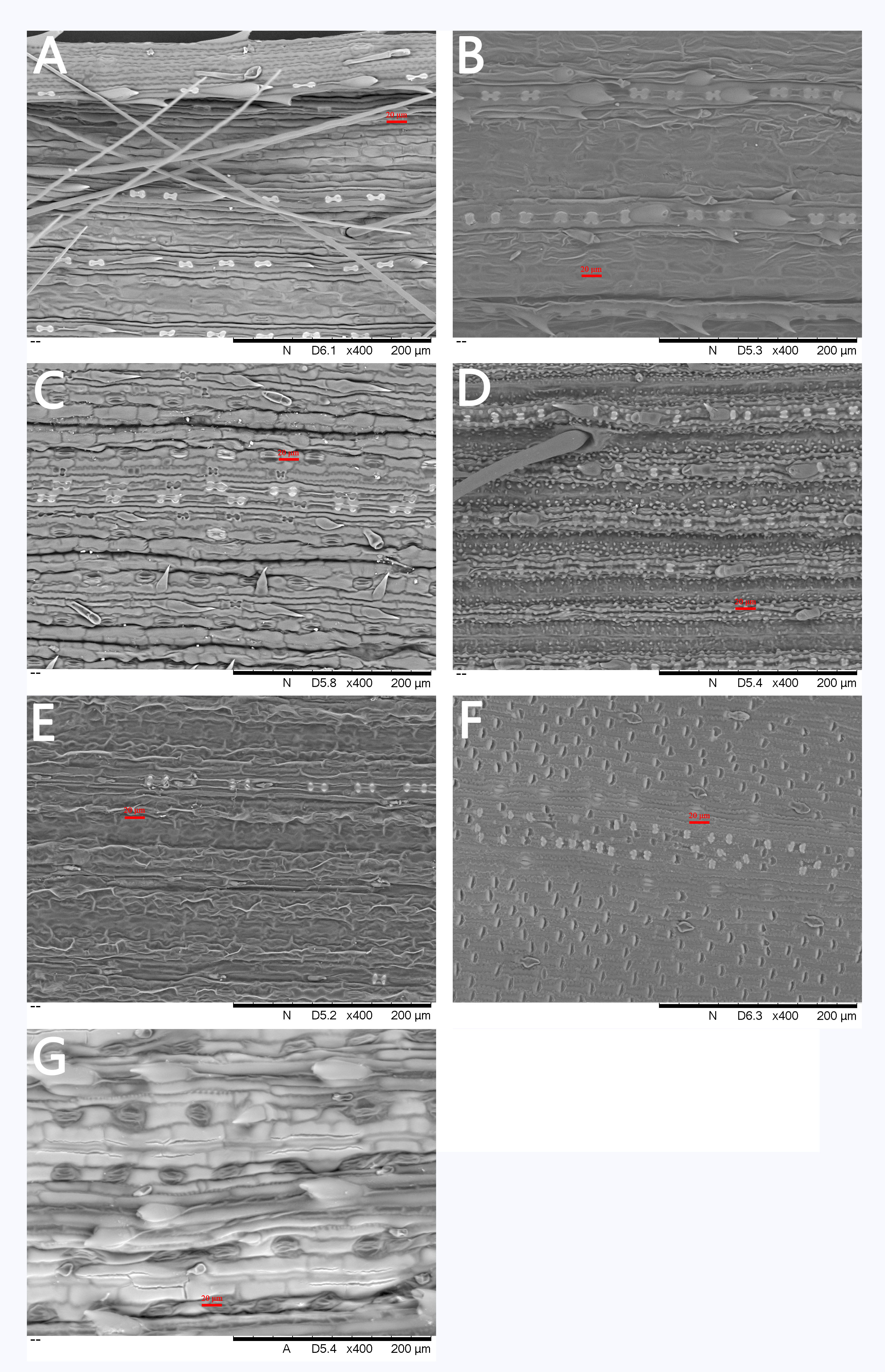

Supplement: Supplementary Figure 6 — Microhair type found in Chloridoideae. (A) enneapogonoid type, (B) long-base cell microhair with sharp-cap cell, (C) long-base cell microhair with round-cap cell, (D) long-base cell microhair with non-constricted base, (E)narrow equal-base-cell microhair, (F)well-proportioned equal-base-cell microhair with sharp-cap cell, (G)short equal-base-cell microhair with round-cap cell. Red scale bars: 20 μm. [file Image_6.jpeg]

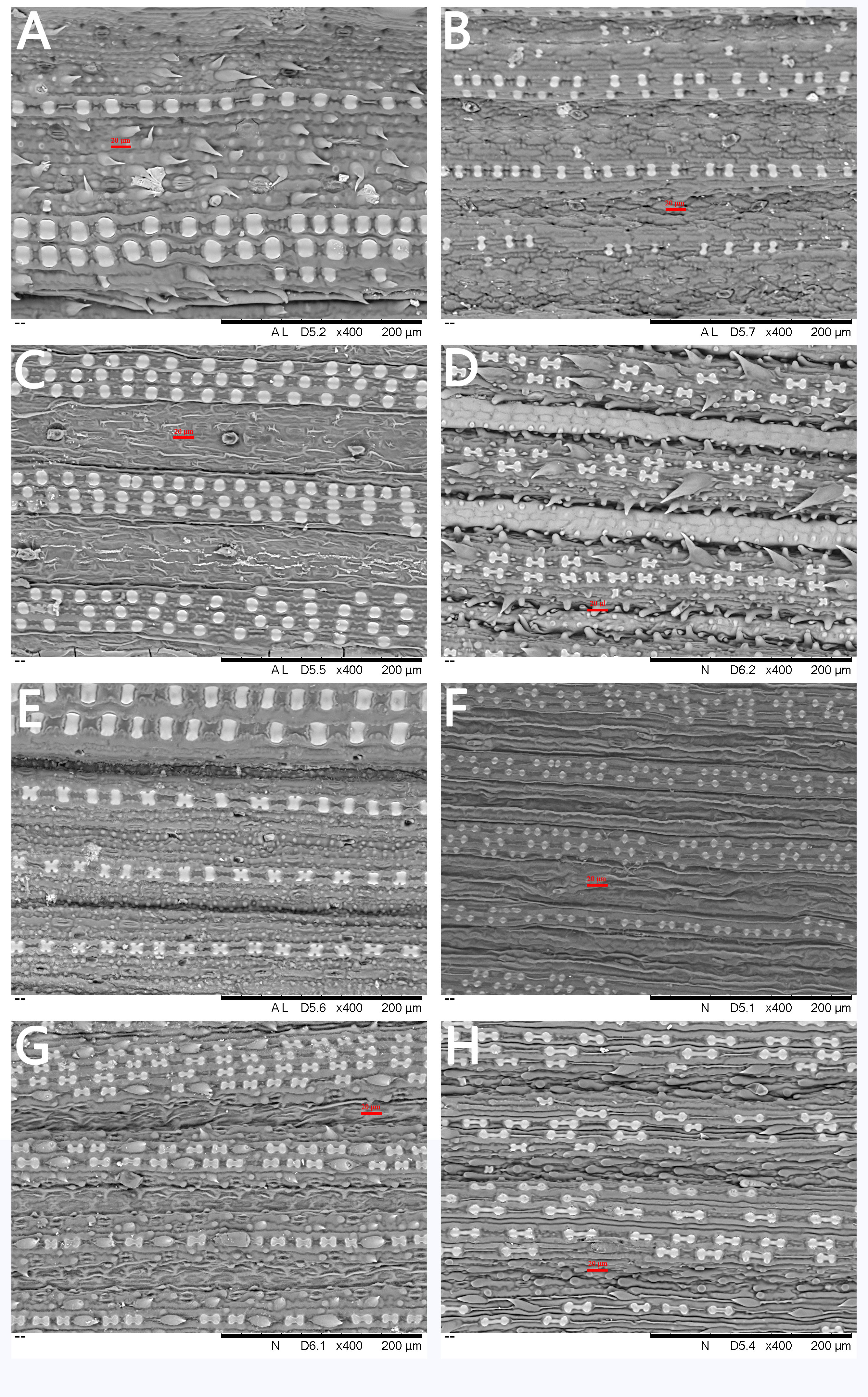

Supplement: Supplementary Figure 7 — Phytoliths found in Chloridoideae. (A) square, (B) saddle, (C) oval, (D) short dumbbell type with square lobes, (E) four-lobes, cross, (F) two-lobed with no obvious rod-like structure, (G) short dumbbell type with round lobes, (H) long dumbbell type with round lobes. Red scale bars: 20 μm. [file Image_7.jpeg]
